# *Novitates neocaledonicae* X: A very rare and threatened new microendemic species of *Acropogon* (Malvaceae, Sterculioideae) from New Caledonia

**DOI:** 10.3897/phytokeys.110.27599

**Published:** 2018-10-26

**Authors:** Gildas Gâteblé, Jérôme Munzinger

**Affiliations:** 1 Institut Agronomique néo-Calédonien (IAC), Equipe ARBOREAL, BP 711, 98810 Mont-Dore, New Caledonia Institut Agronomique néo-Calédonien Mont-Dore New Caledonia (Fr); 2 AMAP, IRD, CIRAD, CNRS, INRA, Université Montpellier, F-34000 Montpellier, France Université Montpellier Montpellier France

**Keywords:** *
Acropogon
*, geology, Malvaceae, New Caledonia, new species, Sterculioideae, taxonomy, threatened species

## Abstract

A new species, *Acropogonhorarius* Gâteblé & Munzinger, **sp. nov.** (Malvaceae, Sterculioideae), is described from New Caledonia. It is known only from two very small subpopulations in the rainforests of the Petchécara Pass between Thio and Canala, in the southeast of Grande-Terre, New Caledonia’s main island. This shrub to small tree has hastate leaves and minute sessile tubular whitish-yellowish flowers and is strikingly different from all other members of the genus. The type locality is geologically complex and located within one of only four amphibolite lenses known in New Caledonia. A line drawing and colour photos are provided for the new species, along with a preliminary risk of extinction assessment, which indicates that the species is Critically Endangered.

## Introduction

**Figure 1.**
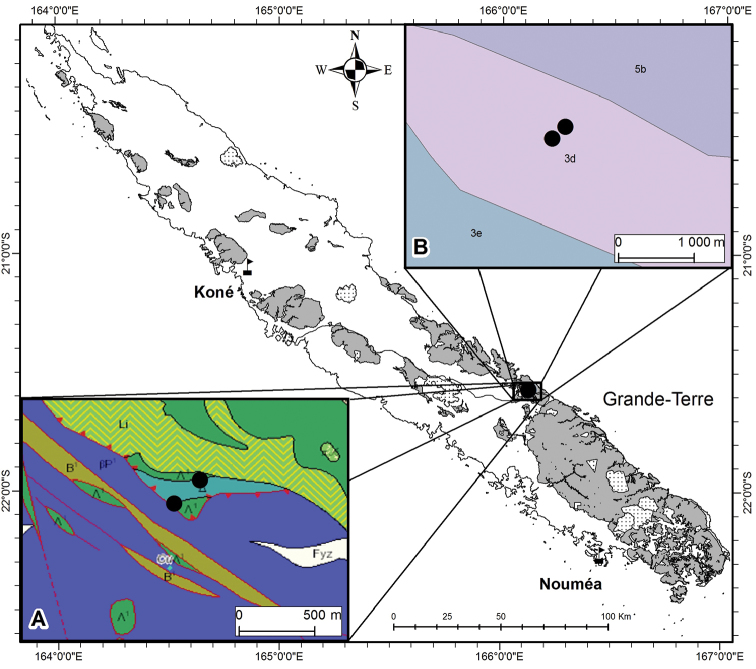
Distribution of *Acropogonhorarius* Gâteblé & Munzinger sp. nov. mapped on the geological (**A**) and soil (**B**) GIS layers of the Petchécara Pass. Insert A, geology from Gouvernement de la Nouvelle-Calédonie (2018): Λ^1^, serpentinites; Δ, amphibolites; B^1^, undifferentiated poly-metamorphic substrate; βP^1^, undifferentiated basalts and dolerites; Fyz, recent and extant alluvial; Li, listwanites. Insert B, soils from Fritsch (2012): 3d, haplic cambisol combined with lithic leptosol on peridotites; 3e, ferralic cambisol combined with haplic ferralsol on volcano-sedimentary and metamorphic rocks; 5b, posic ferralsol on peridotites.

The endemic New Caledonian genus *Acropogon* Schltr. (Malvaceae, Sterculioideae) comprises 26 currently recognised species (Munzinger and Gâteblé 2017). A putatively undescribed species of *Acropogon* was recently found by Jean-Jacques Villegente and Jacqueline Ounémoa in the rainforests of the Petchécara Pass and reported to the first author. This part of New Caledonia, along the locally famous and last remaining “scheduled road” or “route à horaire” along which traffic between the towns of Thio and Canala and above the Dothio river alternates one direction then the other, is not very well known from a botanical perspective. The place where the putative new species grows is also interesting geologically as it is at the edge of a peridotite massif, on one of only four rare amphibolite lenses known in New Caledonia (Cluzel et al. 2012) and close to or within a serpentinite vein according to the geological map of the territory (Gouvernement de la Nouvelle-Calédonie 2018; Fig. 1A). In such places of geological complexity, a large diversity of soils derived from the initial parent material can be found and it is often difficult to characterise the soils precisely (Fritsch 2012; Fig. 1B). However, it is important to conserve these contact zones between soil types to maintain evolutionary processes such as speciation and hybridisation (Pillon et al. 2009).

In this paper, we describe and illustrate a morphologically distinctive new *Acropogon* species. We also provide a risk of extinction assessment based on the IUCN Red List Categories and Criteria (IUCN 2017).

## Materials and methods

In order to determine whether the material from the Petchécara Pass belonged to a currently recognised species or represented a new member of the genus, we carefully examined and measured living specimens and herbarium material. We focused on morphological characters recognised by previous authors (Schlechter 1906; Morat 1986, 1988; Morat and Chalopin 2003, 2005, 2007; Callmander et al. 2015) as being taxonomically informative within the genus. Undetermined herbarium specimens from MPU, NOU and P were studied to assess whether additional material had also been collected previously. The new species was also observed in the field and under cultivation. In order to obtain specimens of the minute and fragile flowers, a fertile branch (cutting) with flower buds was placed in a propagation house (22 °C ± 2 °C, 95% relative humidity) for one month to gather flowers in alcohol as soon as they opened. Descriptions of colour pertain to material seen in vivo, unless otherwise noted.

## Taxonomy

### Acropogon horarius

Taxon classificationPlantaeMalvalesMalvaceae

Gâteblé & Munzinger sp. nov.

urn:lsid:ipni.org:names:77191339-1

Figures 2 , 3

#### Diagnosis.

*Acropogonhorarius* Gâteblé & Munzinger differs from all other members of the genus by the combination of its long and thin petioles, hastate and cordate leaves and minute sessile tubular whitish-yellowish flowers.

#### Type.

New Caledonia. Province Sud: Thio, Col de Petchécara, route à horaire, 200 m alt., 21°34'41.01"S, 166°07'22.41"E, 25 Aug 2016, *G. Gâteblé, J. Ounémoa, M. Moenteapo & E. Poitchili 806* (holotype: P00722668; isotypes: K, MPU311373, NOU088956, P00722670).

**Figure 2.**
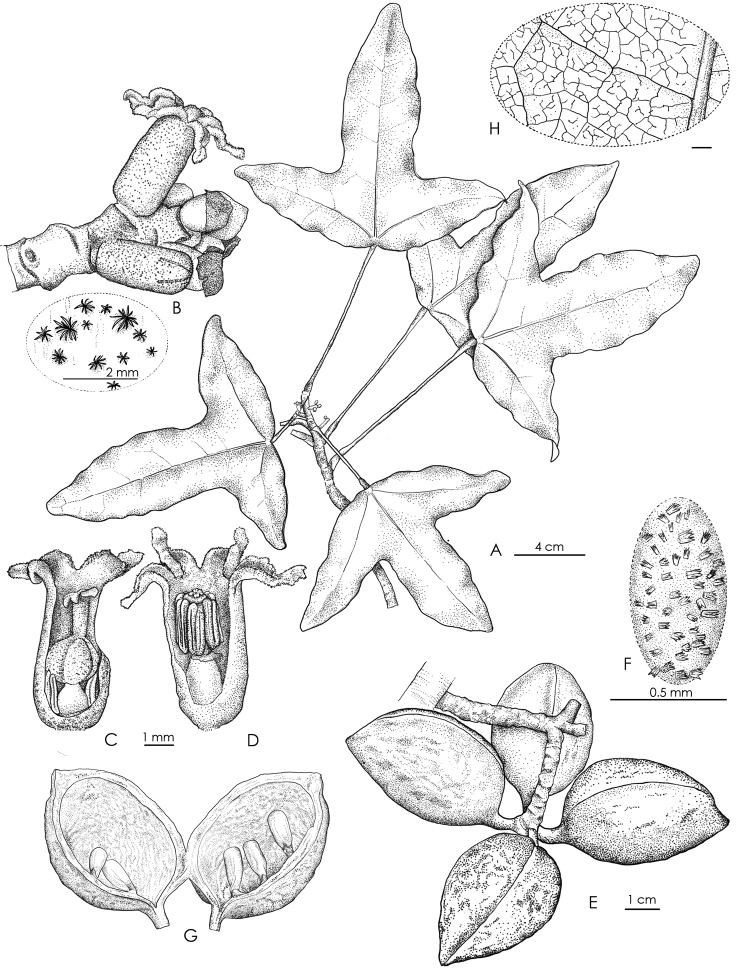
*Acropogonhorarius* Gâteblé & Munzinger sp. nov. **A** flowering branch **B** inflorescence and a zoom on stellate trichomes of the flower tube **C** female flower **D** male flower **E** infructescence **F** detail of erect stellate trichomes on the follicle’s surface **G** detail of an open follicle **H** close-up of the abaxial leaf reticulum. Drawings by Laurence Ramon (A–H *Gâteblé et al. 806*).

#### Description.

Monoecious shrub to small tree up to 6 m tall, sparsely branched with usually a main trunk less than 7 cm in d.b.h.; bark brown, with conspicuous scars left by the caducous cataphylls and sometimes petioles of upper leaves subtending the terminal bud. Leaves clustered at the apex of branches on adult plants, branches 4–5 mm in diameter; petioles light green to yellow (greyish to orange in herbarium material), strongly different in colour than the branch, glabrous, (6.5–)9.0–12.0(–15.5) cm long, 1–2 mm in diameter, elliptical in cross section (in vivo), slightly striate (in herbarium material), enlarged (3–4 mm) proximally and distally, the pulvini slightly pruinose (in vivo); blades simple, coriaceous, flat, slightly discolorous, glabrous on both surfaces, unlobed to slightly hastate on juvenile plants (*Gâteblé et al. 804*), rarely unlobed to strongly trilobed or hastate on adult plants, (10.5–)12.5–15.0(–16.0) cm long, (6.0–)11.5–18.0(–23.5) mm wide, base cordate (rarely truncate), apex of the lobes broadly acute to rounded; generally with 3 strong primary palmate veins, primary and secondary veins prominent abaxially, conspicuously different in colour (yellow-orange in herbarium material and light green to yellowish in vivo) than the blade, reticulum visible abaxially (and adaxially in herb.), secondary veins 2–5 pair; tertiary and quaternary veins finely reticulate with scattered crateriform glands 25–35 µm. Inflorescence a reduced spike-like raceme, axillary within, above or just below the terminal cluster of leaves, up to 3.5 cm long, 2.5–3.5 mm in diameter, axes greenish-yellowish, covered with a dense rust-brown indumentum composed of minute stellate trichomes ca. 90 µm x 90 µm, bracts covered with rust-brown tomentum adaxially. Pedicels minute. Male and female flowers seemingly randomly distributed within the inflorescence, solitary, of the same size. Calyx tubular, 4–5 mm long, 2–2.5 mm in diameter, yellowish and with scattered rust-brown stellate trichomes outside, whitish and glabrous inside, lobes 5, triangular, 1–1.5 mm long, ending in an apical appendage 0.5–1 mm long, interior margins of the lobes and appendages covered with papillose glandular trichomes 35–55 µm long, 15–20 µm in diameter. Male flowers: androecium ca. 4 mm long; androphore tubular, ca. 2 mm long, 0.7–1 mm in diameter, with a few scattered glands; stamens 6–8, ca. 1.7 mm long, inserted at apex of androphore; anther dehiscence longitudinal, extrorse. Female flowers: gynophore ovoid, ca. 1 mm in diameter, with a few scattered glands; staminodes 5–7, composed of sessile, sterile anthers, ca. 1.3 mm long, inserted at the base of the gynophore; ovary ovoid, 1.5–2 mm in diameter, with 3–4 carpels, covered by a dense indumentum of stellate trichomes; style ca. 1 mm long, with scattered stellate trichomes; stigmas 3 or 4 ovoid, 0.4–0.5 mm long. Infructescence 7–12 cm wide, borne on a peduncle 0.5–3 cm long, 2.5–3.5 mm in diameter. Fruit comprising 1–4 follicle(s), each borne on a pedicel 0.5–0.8 cm long, 0.2–0.3 cm in diameter at maturity, green and turning greenish-yellow towards maturity, covered by sparse, erect, stellate trichomes, each follicle ellipsoid to ovoid, 3.5–4.5 cm long, 2.5–3 cm wide, with a woody pericarp ca. 0.2 cm thick in dry material (ca. 0.3 cm in alcohol), apex apiculate. Seeds 4–6 per locule, ellipsoid, white when immature, light brown to black at maturity, 10–12 × 4–6 mm in diameter.

#### Distribution and ecology.

The new species is only known from the south-eastern part of the Grande-Terre, at the Petchécara Pass between Thio and Canala (Fig. 1), where it grows on slopes in rainforest on a soil of complex geological origin.

#### Cultivation note.

Young plantlets of *Acropogonhorarius* are fairly easy to grow in nursery conditions. Shrubs have been grown in a private nursery (Eriaxis nursery) and at the research station of Institut Agronomique néo-Calédonien located in Saint-Louis, Mont-Dore.

#### Etymology.

The plant is named after the last of New Caledonia’s scheduled roads or “route à horaire” along which it grows.

**Figure 3.**
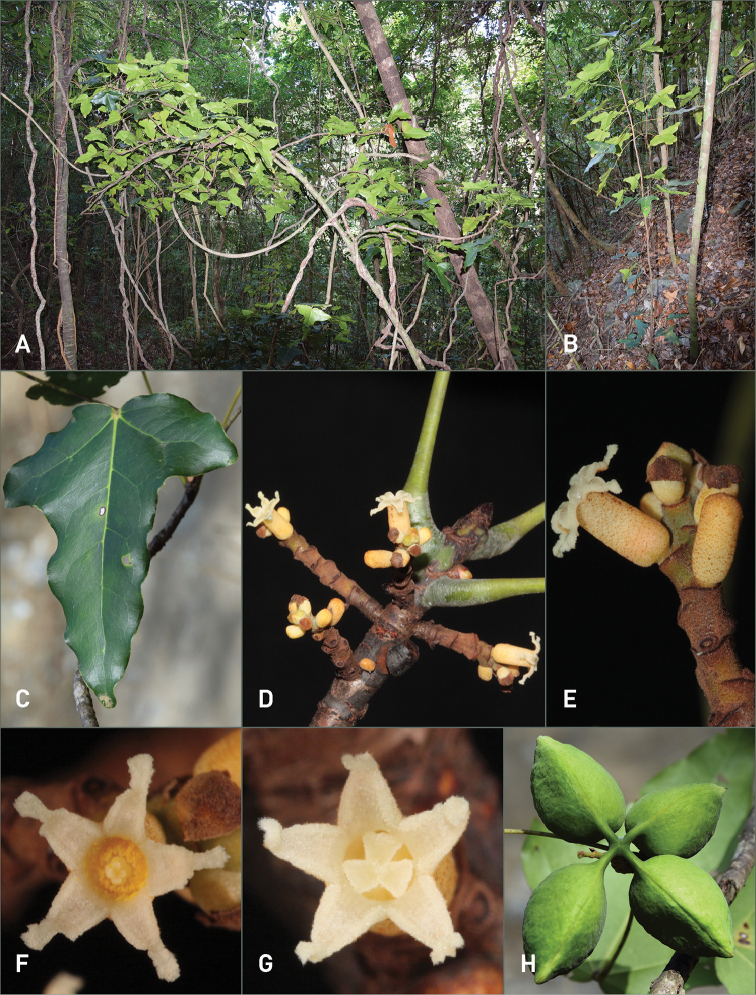
*Acropogonhorarius* Gâteblé & Munzinger sp. nov. **A** overview of a single mature shrub in habitat **B** juvenile plant **C** leaf **D** flowering branch **E** inflorescence **F** male flower **G** female flower **H** follicles. Photographs by G. Gâteblé (**A***Gâteblé et al. 803***B***Gâteblé et al. 804***C–H***Gâteblé et al. 806*).

#### Discussion.

With its hastate and cordate leaves and its long and thin petioles, its minute sessile tubular whitish-yellowish flowers, *Acropogonhorarius* cannot be confused with any other member of this endemic genus. In fact, the morphological characters of its inflorescence [reduced spike-like raceme of solitary (sub)sessile flowers versus true racemes and panicles in other species] and flowers (tubular versus cup shaped in the other taxa) appear to be unique in the genus. A phylogenetic study would be worthwhile to determine whether *A.horarius* belongs to a distinct clade within the genus. The very slow development of the inflorescence axis (or brachyblast), which takes several months, is also unusual as it appears to produce flowers sequentially throughout its growth, perhaps until there is a successful fruit set or until the axis becomes too long (3.5 cm) to produce more flowers, as opposed to the much more nearly synchronous flowering in all other species. The combination of a prolonged duration of flowering and the unusual morphology of the minute tubular flowers suggests that the breeding mechanism in *A.horarius* may be different from that of other members of the genus.

#### Preliminary conservation status

(IUCN 2017). *Acropogonhorarius* is known from only two very small subpopulations on both sides of the road from Thio to Canala and distant from each other by less than 300 m. In the upper subpopulation, about 10 mature individuals were seen whereas only 5 were recorded in the lower subpopulation. During fieldwork, invasive deer (*Rusatimorensis*) were observed in the area and regeneration on the dry forest floor of the upper population is very poor. Deer do not seem to graze the leaves of this *Acropogon* but the habitat is threatened by erosion and reduced regeneration. The lower subpopulation does not seem to be as badly impacted by deer as regeneration is better there, erosion is lower and the forest floor is more moist. However, in the lower subpopulation some rat predation of immature fruits and seeds was observed directly on the trees (see predation marks on fruits of *Gâteblé et al. 805*) and black rat (*Rattusrattus*) predation of fruits was recently reported to be a threat for most *Acropogon* species (Munzinger and Gâteblé 2017). Inasmuch as the two subpopulations are so close to each other, they can be considered as a single location with respect to the main threat (deer). With an EOO and AOO respectively much smaller than 100 km^2^ and 10 km^2^ and with a projected decline in habitat quality, number of subpopulations and mature trees, the species qualifies as Critically Endangered for the IUCN criterion B. Additionally, given that fewer than 50 mature individuals are known, this *Acropogon* also qualifies as CR under criterion C and also under criterion D when combined with a predicted decline in the number of mature individuals. *Acropogonhorarius* is therefore assigned a preliminary status of Critically Endangered, CR B1ab(iii,iv,v)+2ab(iii,iv,v), C1+2a(i), D, based on the IUCN Red List Categories and Criteria (IUCN 2017).

#### Additional specimens examined.

NEW CALEDONIA. Province Sud: Thio, Col de Petchécara, route à horaire, 200 m alt., 21°34'41.01"S, 166°07'22.41"E, 25 Aug 2016, *G. Gâteblé, J. Ounémoa, M. Moenteapo & E. Poitchili 805* (MPU311374, NOU088958, P00722669), Ibid., 270 m alt., 21°34'36"S, 166°07'28"E, 25 Aug 2016, *G. Gâteblé, J. Ounémoa, M. Moenteapo & E. Poitchili 804* (NOU088959, P00722672), Ibid., 25 Aug 2016, *G. Gâteblé, J. Ounémoa, M. Moenteapo & E. Poitchili 803* (K, MEL, MPU311375, NOU088960, P00722671), Ibid., 200 m alt., 21°34'41.01"S, 166°07'22.41"E, 3 Aug2017, *G. Gâteblé & J. Taramoin 981* (MO, MPU311376, NOU088957, P00722674).

## Supplementary Material

XML Treatment for Acropogon horarius
